# Neuropathic post-COVID pain symptomatology is not associated with serological biomarkers at hospital admission and hospitalization treatment in COVID-19 survivors

**DOI:** 10.3389/fmed.2023.1301970

**Published:** 2023-11-28

**Authors:** César Fernández-de-las-Peñas, Carlos Guijarro, María Velasco-Arribas, Juan Torres-Macho, Ana Franco-Moreno, Andrea Truini, Oscar Pellicer-Valero, Lars Arendt-Nielsen

**Affiliations:** ^1^Department of Physical Therapy, Occupational Therapy, Physical Medicine and Rehabilitation, Universidad Rey Juan Carlos (URJC), Madrid, Spain; ^2^Center for Neuroplasticity and Pain (CNAP), SMI, Department of Health Science and Technology, Faculty of Medicine, Aalborg University, Aalborg, Denmark; ^3^Department of Internal Medicine-Infectious Department, Research Department, Hospital Universitario Fundación Alcorcón, Madrid, Spain; ^4^Department of Medicine, Universidad Rey Juan Carlos (URJC), Madrid, Spain; ^5^Department of Internal Medicine, Hospital Universitario Infanta Leonor-Virgen de la Torre, Madrid, Spain; ^6^Department of Medicine, School of Medicine, Universidad Complutense de Madrid, Madrid, Spain; ^7^Department of Human Neuroscience, Sapienza University, Rome, Italy; ^8^Image Processing Laboratory (IPL), Parc Científic de la Universitat de València, Valencia, Spain; ^9^Mech-Sense, Department of Gastroenterology and Hepatology, Clinical Institute, Aalborg University Hospital, Aalborg, Denmark; ^10^Steno Diabetes Center North Denmark, Clinical Institute, Aalborg University Hospital, Aalborg, Denmark

**Keywords:** COVID-19, neuropathic pain, post-COVID, biomarkers, hospitalization

## Abstract

**Objective:**

Evidence suggests that individuals who had survived to coronavirus disease, 2019 (COVID-19) could develop neuropathic post-COVID pain. This study investigated the association of serological biomarkers and treatments received during hospitalization with development of neuropathic-associated symptoms.

**Methods:**

One hundred and eighty-three (*n* = 183) previously hospitalized COVID-19 survivors during the first wave of the pandemic were assessed in a face-to-face interview 9.4 months after hospitalization. Nineteen serological biomarkers, hospitalization data, and treatment during hospitalization were obtained from medical records. Neuropathic pain symptoms (Self-Report Leeds Assessment of Neuropathic Scale), sleep quality (Pittsburgh Sleep Quality Index), pain catastrophizing (Pain Catastrophizing Scale) and anxiety/depressive levels (Hospital Anxiety and Depression Scale) were assessed.

**Results:**

The prevalence of post-COVID pain was 40.9% (*n* = 75). Fifteen (20%) patients reported neuropathic symptoms. Overall, no differences in hospitalization data and serological biomarkers were identified according to the presence or not of neuropathic-associated symptoms. Patients with post-COVID pain had the highest neutrophil count, and *post hoc* analysis revealed that patients with neuropathic post-COVID associated symptoms had lower neutrophil count (*p* = 0.04) compared with those without neuropathic pain, but differences were small and possible not clinically relevant. No differences in fatigue, dyspnea, brain fog, anxiety or depressive levels, poor sleep, or pain catastrophism between patients with and without neuropathic symptoms were found.

**Conclusion:**

It seems that neuropathic-like post-COVID pain symptoms are not associated with neither of assessed serological biomarkers at hospital admission nor hospitalization treatments received in this cohort of hospitalized COVID-19 survivors.

## 1 Introduction

The presence of long-lasting symptoms after the acute phase of a Severe Acute Respiratory Syndrome Coronavirus 2 (SARS-CoV-2) infection has received different terms, being long-COVID or post-COVID-19 condition the most used. The term long-COVID was firstly used and has been proposed for describing the presence of any type of post-COVID symptom (1). The term post-COVID-19 condition was defined, in a Delphi study, as “condition occurring in people with a history of probable or confirmed SARS-CoV-2 acute infection, usually 3 months from the onset of COVID-19 with symptoms that last for at least 2 months after and cannot be explained by an alternative medical diagnosis” (2).

There is a plethora of post-COVID symptoms, and more than 100 symptoms can be attributed to a SARS-CoV-2 infection (3). Current evidence has shown that up to 50% of subjects who had surpassed coronavirus disease, 2019 (COVID-19) could develop any post-COVID symptom the following months (4, 5) and up to one (6, 7) or two (8) years after the infection. Pain is a prevalent post-COVID symptom after fatigue, dyspnea and brain fog (4–8). Two meta-analyses specifically investigating the prevalence of post-COVID pain reported prevalence rates ranging from 5 to 20% for different post-COVID pain symptoms such muscle pain, joint pain or chest pain (9, 10). These rates are based on studies investigating post-COVID pain in an overall post-COVID-19 condition (9, 10). However, studies specifically investigating post-COVID pain reported prevalence rates from 40 to 60% (11–14). Thus, pain could be an underestimated post-COVID symptom.

Clinical characterization of post-COVID pain has received particularly attention. Although post-COVID pain has primarily been described as musculoskeletal phenotype, neuropathic pain has also been described as a potential post-COVID sequelae (15). Single studies have reported that neuropathic-associated pain symptoms can be present in 20–25% of patients with post-COVID pain (16, 17). A recent meta-analysis reported a pooled prevalence of post-COVID neuropathic pain of 34.3% (95%CI, 14.3–62%) (18). However, data can be difficult to compare as the symptoms have been assessed at different time periods after the infection.

Identification of factors associated with neuropathic post-COVID pain could help for timely and personalized treatment interventions and provide evidence to convey to patients. Williams and Zis did not find studies investigating any biomarker potentially associated with neuropathic post-COVID pain risk (18). Magdy et al. found preliminary evidence showing that neuropathic post-COVID pain can be associated with serum levels of neurofilament light chain (NFL) as a potential biomarker (19). The role of serological biomarkers in post-COVID neuropathic-associated pain is scarce (20). In fact, most research on serological biomarkers have mainly focused on the acute phase of the infection. Thus, it has been found that patients with severe COVID-19 exhibit higher C-reactive protein (CRP), D-Dimer and lactate dehydrogenase (LDH) levels, but lower albumin levels than those with non-severe COVID-19 (21).

Increasing evidence has been conducted on the role of serological biomarkers and post-COVID symptomatology. A recent meta-analysis pooling data from 24 biomarkers identified that subjects with post-COVID symptomatology exhibit higher levels of CRP, D-dimer, LDH and leukocytes than those without post-COVID symptoms (22). However, sensitivity analyses by groups of patients revealed that these biomarker changes could be symptom-specific (22). Similar findings have also been reported by the review of Li et al. who concluded that some specific biomarkers can be associated with specific post-COVID symptoms (23).

The association between serological biomarkers and post-COVID pain is also heterogeneous. Bakılan et al. observed lower lymphocyte count but higher D-dimer levels at hospital admission in people with post-COVID pain of musculoskeletal origin (24). Fernández-de-las-Peñas et al. found that patients with musculoskeletal post-COVID pain showed higher lymphocyte count but lower glucose and creatine kinase levels at hospital admission than those without post-COVID pain 8 months after the infection (25). These discrepancies have been identified in a recent meta-analysis investigating biomarkers and post-COVID pain (10).

A previous study found that the presence of neuropathic-associated post-COVID symptoms was not associated with several serological biomarkers at hospital admission (26). This study included a small sample, just COVID-19 survivors with post-COVID pain (neuropathic or not) but did not control for hospitalization treatments which may also lead to neuropathic-like symptomatology. Williams and Zis found preliminary evidence suggesting that the use of some medications, e.g., azithromycin, during hospitalization could be associated with development of COVID-19-related neuropathic pain (18). If an association between serological biomarkers at acute COVID-19 and hospitalization treatments with neuropathic post-COVID pain is identified, therapeutic interventions targeting these mechanisms could be applied to patients during the hospitalization phase as preventing treatment strategy.

The current study investigated (1) if serological biomarkers at hospital admission and treatments received during hospitalization are associated with the development of post-COVID neuropathic-associated pain symptoms in previously hospitalized COVID-19 survivors and (2) if COVID-19 survivors with neuropathic-like pain symptoms exhibit more frequent or more severe concomitant post-COVID (e.g., fatigue, dyspnea or brain fog) or psychological (e.g., anxiety or depressive levels, poor sleep quality) symptoms compared with those without neuropathic-associated pain symptomatology.

## 2 Methods

### 2.1 Participants

Patients hospitalized during the first wave of the pandemic (March–May 2020) at an urban hospital in Spain due to SARS-CoV-2 infection were invited to participate. To be included, patients should have been diagnosed with SARS-CoV-2 infection by real-time reverse transcription-polymerase chain reaction (PCR) assay of nasopharyngeal/oral swab sample and the presence of clinical and radiological findings at hospital admission. The study was approved by the Local Ethic Committee of the hospital (HUFA20/126). Participants were informed of the study and all provided their respective informed consent at the time of the interview.

### 2.2 Hospitalization data

Data about COVID-19 associated onset symptoms at hospital admission, previous comorbidities, and days at hospital were systematically collected at hospitalization. Thus, serological values of the following biomarkers: CPR, leucocyte count, neutrophil count, lymphocyte count, LDH, creatine kinase (CK), alanine transaminase (ALT), aspartate transaminase (AST), urea, hematocrit, creatine phosphokinase (CPK), platelet count, D-dimer, albumin, bilirubin, glucose, sodium, potassium levels as well as activated partial thromboplastin time (APTT) were also systematically collected from blood sample of each participant at hospital admission.

Further, treatments received during hospitalization were also collected from the following list: oral corticoids, inhaled corticoids, statins, antiaggregant, anticoagulant, non-steroidal anti-inflammatory drugs (NSAIDs), heparin, antibiotics, Tocilizumab, Lopinavir/Ritonavir, Hydroxychloroquine, Methylprednisone, or Dexamethasone.

### 2.3 Collection data

A personal face-to-face appointment conducted by trained healthcare researchers was scheduled for collecting data. Participants were asked for the presence of four post-COVID symptoms: pain, fatigue, dyspnea at exertion, and brain fog. They were asked for those symptoms starting after hospital discharge and whether the reported pain persisted at the time of the study. We defined post-COVID pain as: (1) symptoms compatible with chronic primary musculoskeletal pain according to the International Association for the Study of Pain (IASP) (27); (2) symptoms experienced for at least three consecutive months after hospital discharge, and (3) absence of any potential underlying medical condition which could best explain pain, e.g., arthritis. We did not include headache due to the need for a diagnosis according to agreed classifications. In those patients reporting the presence of pain symptoms, a numerical pain rating scale (NPRS, 0–10 points) was used to assess the intensity of the pain.

Additionally, a structured questionnaire including several patient-reported outcome measures (PROMs) evaluating neuropathic pain features (Self-Report Leeds Assessment of Neuropathic Scale, S-LANSS) (28), anxiety and depressive levels (Hospital Anxiety and Depression Scale, HADS) (29), sleep quality (Pittsburgh Sleep Quality Index, PSQI) (30) and pain catastrophism (Pain Catastrophizing Scale, PCS) (31) was used.

The S-LANSS uses a binary response where patients confirm whether they suffer from symptoms that could classify if their pain present a predominant or non-predominant neuropathic origin. The total score ranges from 0 to 24 points, where a cut-off ≥12 points suggest the presence of neuropathic symptoms (28). The S-LANSS has shown proper sensitivity, good internal consistency and validity for identifying neuropathic symptoms (28).

The HADS includes 7 items assessing anxiety symptoms (HADS-A, 21 points) and 7 items assessing depressive symptoms (HADS-D, 21 points) (29). A cut-off score of ≥8 points on each scale has shown good sensitivity and specificity (32). The HADS has shown good validity in people with long-COVID symptomatology (33).

The PSQI evaluates sleep quality over the previous month by including 19 self-rated questions about usual bed-time, usual wake time, number of hours slept, and number of minutes to fall asleep and answered on a 4-point Likert-type scale (0–3). The total score ranges from 0 to 21 points. A cut-off score ≥ 8 points is indicative of poor sleep quality (30). The PSQI has shown good internal consistency and reliability (34).

The PCS includes 13-items evaluating rumination, magnification, and despair aspects in relation to the subject’s pain experience. The responses are rated from 0 (never) to 4 (always) leading to a score ranging from 0 to 52 points (31).

### 2.4 Statistical analysis

Data analysis was conducted with STATA 16.1 program (StataCorp. 2019. Stata Statistical Software: Release 16. College Station, TX: StataCorp LP. USA). Missing values were imputed by using median imputation. The Shapiro–Wilk test was used to assess the assumption of normality and when appropriate to use nonparametric tests. First, McNemar’s chi-squared test and paired Student t-tests were used to compare proportions and means between patients with and without post-COVID pain (without differentiation between neuropathic and non-neuropathic pain). Second, a one-way ANOVA was used to compare all variables between patients without post-COVID pain, patients with post-COVID pain and S-LANSS<12 points, and patients with post-COVID pain and S-LANSS ≥12 points. For all inferences, a corrected *p*-value of <0.05 was considered significant.

## 3 Results

### 3.1 Participants

From 220 patients screened for eligibility, finally, 183 (83%) fulfilled all criteria, agreed to participate, and all serological biomarkers and hospitalization treatment data were obtained. Participants were assessed a mean of 9.4 ± 3.4 months after hospitalization.

### 3.2 Comparison between patients with and without post-COVID pain

The prevalence of post-COVID pain in the sample was 40.9% (*n* = 75). Table 1 summarizes clinical and hospitalization data of COVID-19 survivors depending on the presence or absence of post-COVID pain. No significant differences in demographic data (sex: *p* = 0.742; age: *p* = 0.508; weight: *p* = 0.862; height: *p* = 0.903), pre-existing medical co-morbidities (number medical comorbidities: *p* = 0.534; hypertension: *p* = 0.754; obesity: *p* = 0.240; cardiovascular diseases: *p* = 0.688; chronic obstructive pulmonary disease: *p* = 0.340; asthma: *p* = 0.475), pre-existing pain symptoms (*p* = 0.988), and COVID-19 onset symptoms at hospital admission (number symptoms: *p* = 0.612; fever: *p* = 0.708; dyspnea: *p* = 0.871; myalgias: *p* = 0.635; cough: *p* = 0.599; headache: *p* = 0.533; diarrhea: *p* = 0.748; anosmia: *p* = 0.730; ageusia: *p* = 0.261; throat pain: *p* = 0.245; vomiting: *p* = 0.316; dizziness: *p* = 0.224) and hospital stay (*p* = 0.401). The only difference was that a greater proportion of patients with pre-existing diabetes developed post-COVID pain (*p* = 0.02).

**Table 1:** Clinical and hospitalization data according to the presence or absence of post-COVID pain.

	No post-COVID pain (*n* = 108)	Post-COVID pain (*n* = 75)
Age, mean (SD), years	56.5 (13.5)	57.9 (11.0)
Male/female (%)	57 (52.8%) / 51 (47.2%)	37 (49.3%) / 38 (50.7%)
Weight, mean (SD), kg	81.3 (15.9)	80.9 (16.5)
Height, mean (SD), cm	167.35 (9.25)	167.5 (9.6)
Number of medical comorbidities	1.35 (1.0)	1.3 (1.1)
Medical co-morbidities		
Hypertension Obesity Asthma Cardiovascular diseases Diabetes* Chronic obstructive pulmonary disease	39 (36.1%) 38 (35.2%) 13 (12.05%) 9 (8.3%) 5 (4.6%) 4 (3.7%)	25 (33.3%) 19 (25.3%) 12 (16.0%) 5 (6.7%) 11 (14.7%) 1 (1.3%)
Previous pain symptomatology, *n* (%)	52 (48.15%)	36 (48.0%)
Number of COVID-19 symptoms at hospital admission, mean (SD)	3.4 (0.8)	3.3 (0.9)
Symptoms at hospital admission, *n* (%)		
Fever Dyspnea Myalgias Cough Headache Diarrhea Anosmia Ageusia Throat pain Vomiting Dizziness	86 (79.6%) 39 (36.1%) 59 (54.6%) 38 (35.2%) 39 (36.1%) 24 (22.2%) 26 (24.1%) 29 (26.8%) 15 (13.9%) 8 (7.4%) 4 (3.7%)	56 (74.7%) 26 (34.7%) 45 (60.0%) 30 (40.0%) 23 (30.7%) 15 (20.0%) 20 (26.7%) 14 (18.7%) 6 (8.0%) 9 (12.0%) 6 (8.0%)
Stay at the hospital, mean (SD), days	7.9 (6.7)	9.1 (12.1)
Treatments received at hospitalization, *n* (%)		
Oral corticoids Inhaled corticoids Statins Antiaggregant Anticoagulants NSAIDs Oxigen therapy (no intubation) Lopinavir/ritonavir Hydroxychloroquine Antibiotics Tocilizumab Methylprednisone Dexamethasone Heparine	7 (6.5%) 10 (9.25%) 22 (20.4%) 10 (9.25%) 17 (15.75%) 19 (17.6%) 36 (33.3%) 24 (22.2%) 57 (52.8%) 51 (47.2%) 6 (5.55%) 9 (8.3%) 3 (2.8%) 62 (57.4%)	7 (9.3%) 13 (17.3%) 24 (32.0%) 6 (8.0%) 9 (12.0%) 17 (22.7%) 34 (45.3%) 17 (22.7%) 47 (62.7%) 33 (44.0%) 3 (4.0%) 7 (9.3%) 2 (2.7%) 47 (62.7%)

n, number; SD, Standard Deviation; *Statistically significant differences between groups (*p* < 0.01).

Additionally, no significant differences in hospitalization treatment received were either observed according to the development or not of post-COVID pain (Table 1): oral corticoids (*p* = 0.493), inhaled corticoids (*p* = 0.129), antiaggregant (*p* = 0.776), heparin (*p* = 0.650), statins (*p* = 0.123), anticoagulants (*p* = 0.509), NSAIDs (*p* = 0.446), antibiotics (*p* = 0.751), oxygen therapy without intubation (*p* = 0.201), Hydroxychloroquine (*p* = 0.382), Tocilizumab (*p* = 0.640), Methylprednisone (*p* = 0.822), Dexamethasone (*p* = 0.964), and/or Lopinavir/ Ritonavir (*p* = 0.951).

Patients suffering from post-COVID pain exhibited lower LDH levels (*p* = 0.01) and higher neutrophil count (*p* = 0.04) than those without post-COVID pain (Table 2). No differences between patients with/without post-COVID pain symptoms for the remaining biomarkers were observed: CPR (*p* = 0.361), CPK (*p* = 0.329), APTT (*p* = 0.462), leucocyte count (*p* = 0.228), lymphocyte count (*p* = 0.267), CK (*p* = 0.313), ALT (*p* = 0.294), AST (*p* = 0.153), bilirubin (*p* = 0.938), glucose (*p* = 0.447), hematocrit (*p* = 0.21), platelet count (*p* = 0.998), D-dimer (*p* = 0.376), albumin (*p* = 0.280), urea (*p* = 0.576), sodium (*p* = 0.248) and potassium (*p* = 0.858) levels (Table 2).

**Table 2:** Laboratory biomarkers according to the presence or absence of post-COVID pain.

	No post-COVID pain (*n* = 108)	Post-COVID pain (*n* = 75)
C reactive protein	73.33 (54.38)	81.05 (58.18)
Lactate dehydrogenase (LDH, U/L)*	294.12 (81.91)	266.13 (69.84)
Creatine phosphokinase (CPK, mcg/L)	128.22 (94.08)	166.94 (396.31)
Activated partial thromboplastin time (APTT, seconds)	31.94 (5.34)	31.32 (5.92)
Leucocytes (x10^9^/L)	6.12 (1.72)	6.48 (2.32)
Neutrophils (x10^9^/L)*	4.29 (1.62)	4.77 (1.99)
Lymphocytes (x10^9^/L)	1.3 (0.46)	1.2 (0.55)
Creatine kinase (CK, mg/dL)	0.89 (0.21)	0.96 (0.59)
Alanine transaminase (ALT, U/L)	41.45 (24.05)	37.44 (27.05)
Aspartate transaminase (AST, U/L)	43.15 (20.69)	38.49 (22.92)
Bilirubin (mg/dL)	0.57 (0.24)	0.57 (0.20)
Glucose (mg/mL)	109.73 (30.95)	112.82 (19.92)
Hematocrit (%)	44.88 (3.17)	43.81 (3.92)
Platelets (x10^9^/L)	227.92 (86.28)	227.89 (69.19)
D-dimer (ng/mL)	1022.57 (984.66)	900.07 (813.70)
Albumin (g/dL)	4.53 (0.32)	4.58 (0.28)
Urea (mg/dL)	32.45 (10.84)	33.74 (20.17)
Sodium (mEq/L)	138.76 (3.02)	138.27 (2.48)
Potasium (mmol/L)	4.13 (0.44)	4.12 (0.40)

n, number; SD, Standard Deviation; *Statistically significant differences between groups (*p* < 0.05).

### 3.3 Presence of neuropathic post-COVID associated-pain symptoms

From 75 patients reporting post-COVID pain, fifteen (20%) exhibited neuropathic post-COVID symptoms (S-LANSS score 21.5, SD 5.5). No differences in demographic data (sex: *p* = 0.918; age: *p* = 0.592; weight: *p* = 0.819; height: *p* = 0.264), pre-existing co-morbidities (number comorbidities: *p* = 0.702; hypertension: *p* = 0.854; diabetes: *p* = 0.167; obesity: *p* = 0.46; asthma: *p* = 0.355; cardiovascular diseases: *p* = 0.922; chronic obstructive pulmonary disease: *p* = 0.596), pre-existing pain symptoms (*p* = 0.946), and COVID-19 onset symptom at hospital admission (number of symptoms: *p* = 0.879; fever: *p* = 0.901; dyspnea: *p* = 0.559; myalgias: *p* = 0.893; cough: *p* = 0.778; headache: *p* = 0.807; diarrhea: *p* = 0.781; anosmia: *p* = 0.942; ageusia: *p* = 0.299; throat pain: *p* = 0.158; vomiting: *p* = 0.454; dizziness: *p* = 0.459) and days at hospital (*p* = 0.554, Table 3) were identified.

**Table 3:** Clinical and hospitalization data according to the presence or absence of neuropathic-associated symptoms (S-LANSS ≥12 points).

	No post-COVID pain (*n* = 108)	S-LANSS <12 points (*n* = 60)	S-LANSS ≥12 points (*n* = 15)
Age, mean (SD), years	56.5 (13.5)	57.3 (10.8)	60.1 (10.5)
Gender, male/female (%)	57 (52.8%) / 51 (47.2%)	29 (48.3%) / 31 (51.7%)	10 (53.3%) / 7 (46.7%)
Weight, mean (SD), kg	81.3 (15.9)	80.0 (15.0)	84.5 (22.2)
Height, mean (SD), cm	167.35 (9.25)	166.6 (9.9)	171.1 (7.5)
Number of medical comorbidities	1.35 (1.0)	1.25 (1.1)	1.40 (1.2)
Medical co-morbidities			
Hypertension Obesity Asthma Cardiovascular diseases Diabetes Chronic obstructive pulmonary disease	39 (36.1%) 38 (35.2%) 13 (12.05%) 9 (8.3%) 5 (4.6%) 4 (3.7%)	21 (35.0%) 16 (26.7%) 8 (13.3%) 4 (6.7%) 7 (11.7%) 1 (1.7%)	4 (26.7%) 3 (20.0%) 4 (26.7%) 1 (6.7%) 4 (26.7%) 0 (0.0%)
Previous pain symptomatology, *n* (%)	52 (48.15%)	28 (46.7%)	8 (53.3%)
Number of COVID-19 symptoms at hospitalization, mean (SD)	3.4 (0.8)	3.3 (0.85)	3.4 (1.0)
Symptoms at hospital admission, *n* (%)			
Fever Dyspnea Myalgias Cough Headache Diarrhea Anosmia Ageusia Throat pain Vomiting Dizziness	86 (79.6%) 39 (36.1%) 59 (54.6%) 38 (35.2%) 39 (36.1%) 24 (22.2%) 26 (24.1%) 29 (26.8%) 15 (13.9%) 8 (7.4%) 4 (3.7%)	44 (73.3%) 23 (38.3%) 36 (60.0%) 23 (38.3%) 18 (30.0%) 11 (18.3%) 16 (26.7%) 13 (21.7%) 3 (5.0%) 8 (13.3%) 5 (8.35%)	12 (80.0%) 3 (20.0%) 9 (60.0%) 7 (46.7%) 5 (33.3%) 4 (26.7%) 4 (26.7%) 1 (6.7%) 3 (20.0%) 1 (6.7%) 1 (6.7%)
Stay at the hospital, mean (SD), days	7.9 (6.7)	9.45 (13.2)	7.6 (5.7)
Treatments received at hospitalization, *n* (%)			
Oral corticoids Inhaled corticoids Statins Antiaggregant Anticoagulants NSAIDs Oxigen therapy (no intubation) Lopinavir/ritonavir Hydroxychloroquine Antibiotics Tocilizumab Methylprednisone Dexamethasone Heparine	7 (6.5%) 10 (9.25%) 22 (20.4%) 10 (9.25%) 17 (15.75%) 19 (17.6%) 36 (33.3%) 24 (22.2%) 57 (52.8%) 51 (47.2%) 6 (5.55%) 9 (8.3%) 3 (2.8%) 62 (57.4%)	5 (8.3%) 9 (15.0%) 18 (30.0%) 4 (6.7%) 5 (8.3%) 15 (25.0%) 28 (46.7%) 15 (25.0%) 38 (63.3%) 26 (43.3%) 2 (3.3%) 6 (10.0%) 2 (3.3%) 38 (63.3%)	2 (13.35%) 4 (26.7%) 6 (40.0%) 2 (13.35%) 4 (26.7%) 2 (13.35%) 6 (40.0%) 2 (13.35%) 9 (60.0%) 7 (45.7%) 1 (6.7%) 1 (6.7%) 0 (0.0%) 9 (60.0%)

n, number; SD, Standard Deviation; *Statistically significant differences between groups (*p* < 0.01).

No significant differences in hospitalization treatment received were either found (Table 3): oral corticoids (*p* = 0.649), inhaled corticoids (*p* = 0.165), heparin (*p* = 0.892), statins (*p* = 0.239), antiaggregant (*p* = 0.708), anticoagulants (*p* = 0.195), NSAID (*p* = 0.494), antibiotics (*p* = 0.937), oxygen therapy/no intubation (*p* = 0.405), Hydroxychloroquine (*p* = 0.675), Tocilizumab (*p* = 0.783), Methylprednisone (*p* = 0.903), Dexamethasone (*p* = 0.782), and/or Lopinavir/Ritonavir (*p* = 0.693).

No overall differences between patients with and without neuropathic post-COVID associated symptoms for serological biomarkers were identified: CPR (*p* = 0.296), CPK (*p* = 0.347), APTT (*p* = 0.688), leucocyte count (*p* = 0.259), lymphocyte count (*p* = 0.280), CK (*p* = 0.384), ALT (*p* = 0.577), AST (*p* = 0.342), bilirubin (*p* = 0.955), glucose (*p* = 0.513), hematocrit (*p* = 0.114), platelet count (*p* = 0.490), D-dimer (*p* = 0.399), albumin (*p* = 0.489), urea (*p* = 0.710), sodium (*p* = 0.287) and potassium (*p* = 0.787) levels (Table 4). *Post hoc* analyses in relation to LDH levels revealed that differences were not significant between patients with and without neuropathic post-COVID associated pain symptoms, whereas neutrophil count was significantly lower (*p* = 0.04) in individuals with neuropathic post-COVID associated symptoms than in those without neuropathic symptoms (Table 4).

**Table 4:** Laboratory biomarkers according to the presence or absence of neuropathic-associated symptoms (S-LANSS ≥12 points).

	No post-COVID pain (*n* = 108)	S-LANSS <12 points (*n* = 60)	S-LANSS ≥12 points (*n* = 15)
C reactive protein	73.33 (54.38)	85.14 (59.02)	64.72 (53.42)
Lactate dehydrogenase (LDH, U/L)*	294.12 (81.91)	267.88 (69.6)	259.13 (72.81)
Creatine phosphokinase (CPK, mcg/L)	128.22 (94.08)	183.4 (440.63)	101.11 (78.42)
Activated partial thromboplastin time (APTT, seconds)	31.94 (5.34)	31.47 (5.69)	30.73 (6.96)
Leucocytes (x10^9^/L)	6.12 (1.72)	6.61 (2.4)	5.96 (1.94)
Neutrophils (x10^9^/L)*	4.29 (1.62)	4.93 (2.04)	4.13 (1.69)
Lymphocytes (x10^9^/L)	1.3 (0.46)	1.18 (0.51)	1.35 (0.69)
Creatine kinase (CK, mg/dL)	0.89 (0.21)	0.98 (0.65)	0.87 (0.18)
Alanine transaminase (ALT, U/L)	41.45 (24.05)	37.45 (28.79)	37.43 (19.35)
Aspartate transaminase (AST, U/L)	43.15 (20.69)	38.9 (24.19)	36.85 (17.51)
Bilirubin (mg/dL)	0.57 (0.24)	0.57 (0.19)	0.59 (0.26)
Glucose (mg/mL)	109.73 (30.95)	111.46 (19.41)	118.25 (21.7)
Hematocrit (%)	44.88 (3.17)	43.71 (3.93)	44.18 (4.0)
Platelets (x10^9^/L)	227.92 (86.28)	233.39 (69.61)	205.89 (65.11)
D-dimer (ng/mL)	1022.57 (984.66)	845.52 (636.8)	1118.29 (1314.14)
Albumin (g/dL)	4.53 (0.32)	4.57 (0.3)	4.61 (0.22)
Urea (mg/dL)	32.45 (10.84)	34.28 (22.03)	31.57 (9.88)
Sodium (mEq/L)	138.76 (3.02)	138.1 (2.47)	138.97 (2.46)
Potasium (mmol/L)	4.13 (0.44)	4.1 (0.42)	4.18 (0.32)

n, number; SD, Standard Deviation; *Statistically significant differences between patients with and without pain (*p* < 0.05).

### 3.4 Other associated symptomatology

Table 5 shows other associated symptoms according to the presence or absence of neuropathic post-COVID associated pain. No differences for the presence of other post-COVID symptoms such as fatigue (*p* = 0.816), brain fog (*p* = 0.966) or dyspnea on exertion (*p* = 0.805), as well as anxiety levels (HADS-A score: *p* = 0.569; cut-off score: *p* = 0.878), depressive levels (HADS-D score: *p* = 0.279; cut-off score: *p* = 0.519), poor sleep (PSQI score: 0.293; cut-off score: *p* = 0.253), pain catastrophism (PCS: *p* = 0.251) or pain intensity (*p* = 0.289) between patients with/without post-COVID pain (with/without neuropathic associated symptomatology) were observed (Table 5).

**Table 5:** Other post-COVID symptomatology according to the presence or absence of neuropathic-associated symptoms (S-LANSS ≥12 points).

	No post-COVID pain (*n* = 108)	S-LANSS <12 points (*n* = 60)	S-LANSS ≥12 points (*n* = 15)
Fatigue, n (%)	60 (55.5%)	38 (63.3%)	9 (60.0%)
Dyspnea at exertion, n (%)	69 (63.9%)	7 (11.7%)	2 (13.3%)
Brain fog, n (%)	16 (14.8%)	8 (13.3%)	2 (13.3%)
HADS-A (0–21), mean (SD)	3.2 (3.6)	3.65 (4.25)	4.1 (3.4)
Anxiety symptoms (HADS-A ≥ 8), n (%)	13 (12.05%)	9 (15%)	2 (13.3%)
HADS-D (0–21), mean (SD)	2.25 (3.1)	3.1 (3.45)	2.2 (3.1)
Depressive symptoms (HADS-A ≥ 8), n (%)	7 (6.5%)	7 (11.7%)	1 (6.7%)
PSQI (0–21), mean (SD)	6.7 (3.9)	5.9 (3.9)	5.5 (3.2)
Poor sleep (PSQI ≥8 points), n (%)	44 (40.75%)	17 (28.4%)	3 (20%)
S-LANSS (0–24), mean (SD)	–	4.0 (3.6)	21.5 (5.5)
PCS (0–52), mean (SD)	–	6.7 (7.8)	10.8 (8.8)
Pain intensity (NPRS, 0–10), mean (SD)	–	5.4 (1.8)	6.1 (1.9)

n, number; SD, Standard Deviation; *Statistically significant differences between groups (*p* < 0.05).

HADS, Hospital Anxiety and Depression Scale; PSQI, the Pittsburgh Sleep Quality Index; S-LANSS, Self-Report Leeds Assessment of Neuropathic Symptoms Scale; PCS, Pain Catastrophizing Scale; NPRS, Numerical Pain Rate Scale.

## 4 Discussion

This study found that 20% of COVID-19 survivors reporting post-COVID pain had neuropathic-like symptoms. Overall, no differences in serological biomarkers at the acute phase of the infection and hospitalization treatments were identified according to the presence/absence of neuropathic-associated symptoms. *Post hoc* analyses revealed that patients with neuropathic-associated symptoms showed lower neutrophil count than those without neuropathic-associated symptoms, but the differences were small. Thus, no differences in the presence of other post-COVID symptoms were either found between those COVID-19 survivors with and without neuropathic-associated symptoms.

The presence of neuropathic-associated symptoms in patients with post-COVID pain has been previously observed (16–18). This study found that almost 20% of patients with post-COVID pain (*n* = 15, 8.2% of the total sample) exhibited neuropathic-associated symptoms as well as central nervous-derived symptoms such as fatigue, brain fog, sleep problems, or psychological disturbances. Thus, these symptoms were not associated with the presence of post-COVID pain or neuropathic-associated symptoms since they were present in all groups of COVID-19 survivors. Our findings agree with current literature supporting that fatigue, dyspnea, brain fog, and pain are the most prevalent post-COVID symptoms (4–8). Thus, the presence of neuropathic symptoms in a subgroup of patients with post-COVID pain could promote a more complex and serious pain phenotype which could render long term complications.

### 4.1 Serological biomarkers at hospital admission

Since neuropathic pain is associated with pain chronification, post-COVID fatigue, motor and sensory deficits, and higher burden (14), early identification of potential risk factors associated with its development are needed. In such a scenario, identification of factors during the acute phase of SARS-CoV-2 infection could help for identifying subjects at a higher risk. Several hospitalization factors, e.g., immobilization, hospital stay, mechanical ventilation, internal care unit (ICU) admission, or isolation have associated with the development of pain symptoms (35); however, evidence on hospitalization risk factors for post-COVID-19 condition is conflicting (36). At hospitalization, serological biomarkers have received attention, particularly in their association with severe COVID-19 illness (21). However, data on serological biomarkers at the acute phase of the infection and post-COVID-19 condition is heterogeneous (22, 23). Thus, some serological biomarkers have been associated with the overall post-COVID-19 condition (22), but increasing evidence suggests that this association seems to be symptom specific.

The association between serological biomarkers and post-COVID pain, as a specific symptom, is also heterogeneous (10). We observed higher neutrophil count in people with post-COVID pain; data not found by previous studies (24, 25). In fact, Bakılan et al. identified lower lymphocyte count (24) whereas Fernández-de-las-Peñas et al. found higher lymphocyte count (25) at hospital admission in individuals with post-COVID pain. Differences in biomarkers analyzed, post-COVID clinical features and follow-up periods could explain these discrepancies. Further, due to the different pain phenotype that post-COVID pain can adopt (37), heterogeneous underlying mechanisms could also explain these discrepancies.

A previous study did not find any serological biomarker associated with neuropathic-associated pain symptoms (26). In fact, the only serological biomarker identified today with neuropathic post-COVID pain has been serum levels of NFL (19). Surprisingly, *post hoc* analyses of our study revealed that patients with neuropathic-associated symptomatology had lower neutrophils count (neutropenia) when compared with those without neuropathic-like symptoms suggesting the immune response may be different between subjects with and without neuropathic-associated pain. Since a neutropenia status, i.e., low neutrophil count, is associated with autoimmunity and repeated infections it is possible that these patients present an unstable neuroinflammatory condition due to COVID-19 where neutrophils are unregulated due to an uncontrolled immune response (38). It is also possible that a long-lasting inflammatory (39) and immune response after the COVID-19 acute phase would lead to nervous system changes and subsequent development of neuropathic-like pain symptoms. Thus, the presence of overall post-COVID symptomatology has been associated with higher neutrophil count, higher neutrophil/leucocyte ratio, fibrinogen and CRP levels 3 months after the infection; however, this study remarked that the association between these serological biomarkers was symptom specific (40). In fact, no association between any biomarker and post-COVID pain was either identified in this study (40).

Based on current evidence, post-COVID pain has a multifactorial genesis where factors related to the pathogen (SARS-CoV-2 associated-factors) intersect with the individual host response (biological and immune responses), as well as with hospitalization (treatment-associated factors) and emotional (COVID-19 outbreak surrounding elements) factors (41). In fact, complexity of post-COVID pain is reflected by the different phenotypes that can be adopted: nociceptive, neuropathic, or nociplastic (37). In fact, it is also possible that this multifactorial genesis and, hence, the role of serological biomarkers and hospitalization treatments could be different between patients with different post-COVID pain phenotype explaining the different therapeutic approaches needed for each phenotype (37).

### 4.2 Treatments during hospitalization

Our study is the first one specifically investigating if treatments received during hospitalization could be associated with the development of neuropathic-associated post-COVID pain symptoms. The procedures for managing the COVID-19 infection and its symptoms have changed during the pandemic and developed from trial-and-error to more validated treatments. For example, NSAIDs for pain management were for a short while suggested contraindicated due to interaction with the ACE2 system, but this suggestion was later rejected (42).

It has been discussed if any of the anti-COVID-19 drugs may show severe side effects such as neurotoxicity, and Hydroxychloroquine or Chloroquine have been suggested to be neurotoxic (43). In the present patient population none of those medications, when administered at hospitalization, were associated with the development of neuropathic-associated post-COVID symptomatology. The retroviral Ritonavir as some patients in the present study received during hospitalization is also known to be neurotoxic (44), but was not specifically coding for the development of neuropathic-like pain symptoms in the present study probably due to the short-term treatment during hospitalization. Furthermore, Ritonavir and Lopinavir have oligodendrocyte toxicity (45). So, it seems that none of the treatments applied in the present study have any impact in neither the development of post-COVID pain in general nor post-COVID neuropathic-like pain symptoms. Nevertheless, current data should be considered as preliminary and confirmed with population-based studies.

### 4.3 Limitations

Current results should be considered according to potential limitations of the study. First, we just included a sample of previously hospitalized patients, accordingly, we do not know if the same results would be observed in non-hospitalized COVID-19 survivors. In fact, all patients had been hospitalized during the first wave of the COVID-19 pandemic (March–May 2020), when the historical strain was predominant. We do not know if different SARS-CoV-2 variants, e.g., Alpha, Delta or Omicron, would lead to similar pattern. Similarly, all patients were infected and hospitalized due to COVID-19 without receiving any vaccine dose. Today, high percentage of the worldwide population has received at least one dose of any SARS-CoV-2 vaccine. We do not know if potential reinfections after vaccination from a different SARS-CoV-2 variant would require hospitalization. Thus, all patients were recruited from a single hospital, accordingly, multi-center studies including subjects from different hospitals should confirm or refute current results. Second, some comparisons were closed to statistically significance, thus, we cannot exclude an error type II due to the sample size. Future studies included larger samples would be needed. Third, we only collect serological biomarkers at hospital admission, accordingly, we do not know if long-lasting inflammation or immune response would be present after. Third, the presence of neuropathic-associated symptoms was based on the use of a self-reported questionnaire (S-LANSS). We do not know if diagnosis of neuropathic pain symptoms by objective assessment would lead to the same results.

## 5 Conclusion

This study found that the presence of neuropathic-associated symptoms is not associated with serological biomarkers at hospital admission and hospitalization treatments received in a sample of previously hospitalized COVID-19 survivors. The presence of other post-COVID symptoms consisting of fatigue, brain fog or dyspnea as well as psychological disturbances was similar between patients with and without post-COVID pain with or without neuropathic-associated symptomatology.

## Data availability statement

The original contributions presented in the study are included in the article/supplementary material, further inquiries can be directed to the corresponding author.

## Ethics statement

The studies involving humans were approved by the Local Ethic Committee of the Hospital (HUFA20/126). The studies were conducted in accordance with the local legislation and institutional requirements. The participants provided their written informed consent to participate in this study.

## Author contributions

CF-d-l-P: Conceptualization, Data curation, Formal analysis, Funding acquisition, Investigation, Methodology, Validation, Visualization, Writing – original draft, Writing – review & editing. CG: Conceptualization, Investigation, Methodology, Project administration, Resources, Software, Validation, Visualization, Writing – original draft, Writing – review & editing. MV-A: Conceptualization, Investigation, Methodology, Project administration, Resources, Software, Validation, Visualization, Writing – original draft, Writing – review & editing. JT-M: Conceptualization, Data curation, Investigation, Methodology, Project administration, Resources, Software, Validation, Visualization, Writing – original draft, Writing – review & editing. AF-M: Conceptualization, Data curation, Investigation, Methodology, Project administration, Resources, Software, Supervision, Validation, Visualization, Writing – review & editing. AT: Data curation, Investigation, Supervision, Validation, Visualization, Writing – review & editing. OP-V: Data curation, Formal analysis, Investigation, Methodology, Project administration, Software, Supervision, Validation, Visualization, Writing – review & editing. LA-N: Conceptualization, Data curation, Funding acquisition, Investigation, Methodology, Project administration, Supervision, Validation, Visualization, Writing – original draft, Writing – review & editing.
